# Supplementary material for: Age at Natural Menopause in Women Living with HIV: A Cross-Sectional Study Comparing Self-Reported and Biochemical Data
Source: Viruses. 2023 Apr 26;15(5):1058. doi: 10.3390/v15051058 (PMC10221766; doi:10.3390/v15051058)
Supplement: Supplementary file 1 [file viruses-15-01058-s001.zip › viruses-2325404-supplementary.pdf]

Appendix

Table S1. Odds of early menopause (<45 years old) based on self-report with biochemical confirmation of menopause status among women living with HIV (14/83) and HIV-negative women (15/92) in the CARMA-ENDO and BCC3 cohorts

	Unadjusted OR (95% CI)	*p*-value	Adjusted OR (95% CI)	*p*-value
**HIV status** (ref: HIV-negative)	1.04 (0.47 to 2.32)	0.92	1.35 (0.47 to 3.88)	0.57
**Income** (ref: ≥20,000 CAD/year)	1.19 (0.52 to 2.69)	0.68	0.63 (0.20 to 1.87)	0.41
**Parity** (per increase in one birth)	1.03 (0.78 to 1.33)	0.83	0.97 (0.69 to 1.34)	0.87
**BMI*** (per one kg/m² increase)	0.99 (0.93 to 1.05)	0.75	0.98 (0.91 to 1.05)	0.53
**Hepatitis C infection** (ref: never)	1.29 (0.55 to 2.91)	0.55	0.39 (0.09 to 1.52)	0.18
**Smoking** (ref: never)				
Past	1.50 (0.46 to 8.57)	0.48	0.61 (0.11 to 2.64)	0.53
Current	**3.20 (1.28 to 8.57)**	**0.02**	2.66 (0.64 to 11.0)	0.17
**Substance use†** (ref: never)	**2.95 (1.31 to 6.76)**	**0.009**	3.60 (0.97 to 14.5)	0.06
**Ethnicity** (ref: White)				
Non-White	1.17 (0.52 to 2.63)	0.71		
**Age at menarche** (per one year increase)	1.15 (0.89 to 1.48)	0.29		

Biochemically confirmed age at menopause was determined based on the age at the last menstrual period and follicle stimulating hormone levels. *BMI=body mass index; †Substance use=history of opioid, crack/cocaine, and/or methamphetamine use.

Table S2. Factors associated with self-report plus biochemically confirmed age at menopause by median regression in women living with HIV (n=66) and HIV-negative women (n=85) in the CARMA-ENDO and BCC3 cohorts who have never used opioids

	Unadjusted β (95% CI)	*p*-value	Adjusted β (95% CI)	*p*-value
**HIV status** (ref: HIV-negative)	-1.00 (-3.04 to 1.04)	0.33	0.93 (-1.17 to 3.04)	0.38
**Income** (ref: ≥20,000 CAD/year)	-2.00 (4.47 to 0.47)	0.11	0.41 (-1.53 to 2.36)	0.68
**Parity** (per increase in one birth)	0.00 (-0.71 to 0.71)	1.00	-0.08 (-0.74 to 0.59)	0.82
**BMI*** (per one kg/m² increase)	0.00 (-0.10 to 0.10	1.00	-0.06 (-0.19 to 0.07)	0.37
**Hepatitis C infection** (ref: never)	-2.00 (04.45 to 0.45)	0.11	-0.40 (-3.04 to 2.25)	0.77
**Smoking** (ref: never)				
Past	0.00 (-1.66 to 1.66)	1.00	-0.87 (-3.12 to 1.39)	0.45
Current	**-3.00 (-4.95 to -1.05)**	**<0.001**	**-2.77 (-5.14 to -0.41)**	**0.02**

**Substance use†** (ref: never)	**-3.00 (-5.80 to -0.20)**	**0.04**	-1.11 (-4.12 to 1.90)	0.47
**Age at menarche** (per one year increase)	0.00 (-0.51 to 0.51)	1.00	-0.06 (-0.67 to 0.55)	0.85
**Ethnicity** (ref: White)				
Non-White	-1.00 (-2.88 to 0.88)	0.29		

Biochemically confirmed age at menopause was determined based on the age at the last menstrual period and follicle stimulating hormone levels. *BMI=body mass index; †Substance use=history of crack/cocaine or methamphetamine use.

Table S3. Association between HIV-related variables and self-report with biochemically confirmed age at menopause by median regression among women living with HIV (n=66) who have never used opioids in the CARMA-ENDO and BCC3 cohorts

	Unadjusted β (95% CI)	*p*-value	Adjusted β (95% CI)	*p*-value
**Nadir CD4 count** (ref: ≥200 cells/ml)				
<200 cells/ml	1.00 (-6.59 to 8.59)	0.79	3.00 (-3.84 to 9.84)	0.37
**Current CD4 count** (ref: ≥500 cells/ml)				
<500 cells/ml	1.00 (-2.53 to 4.53)	0.57	0.00 (-7.35 to 7.35)	1.00
**Highest HIV viral load** (ref: <100,000 copies/µl) ≥100,000 copies/ml	1.00 (-1.93 to 3.93)	0.50	-1.00 (-11.3 to 9.29)	0.84
**Current viral load** (ref: undetectable)				
Detectable	-2.00 (-8.09 to 4.09)	0.51		
**Years lived with HIV** (per on year increase)	0.08 (-0.09 to 0.25)	0.37		

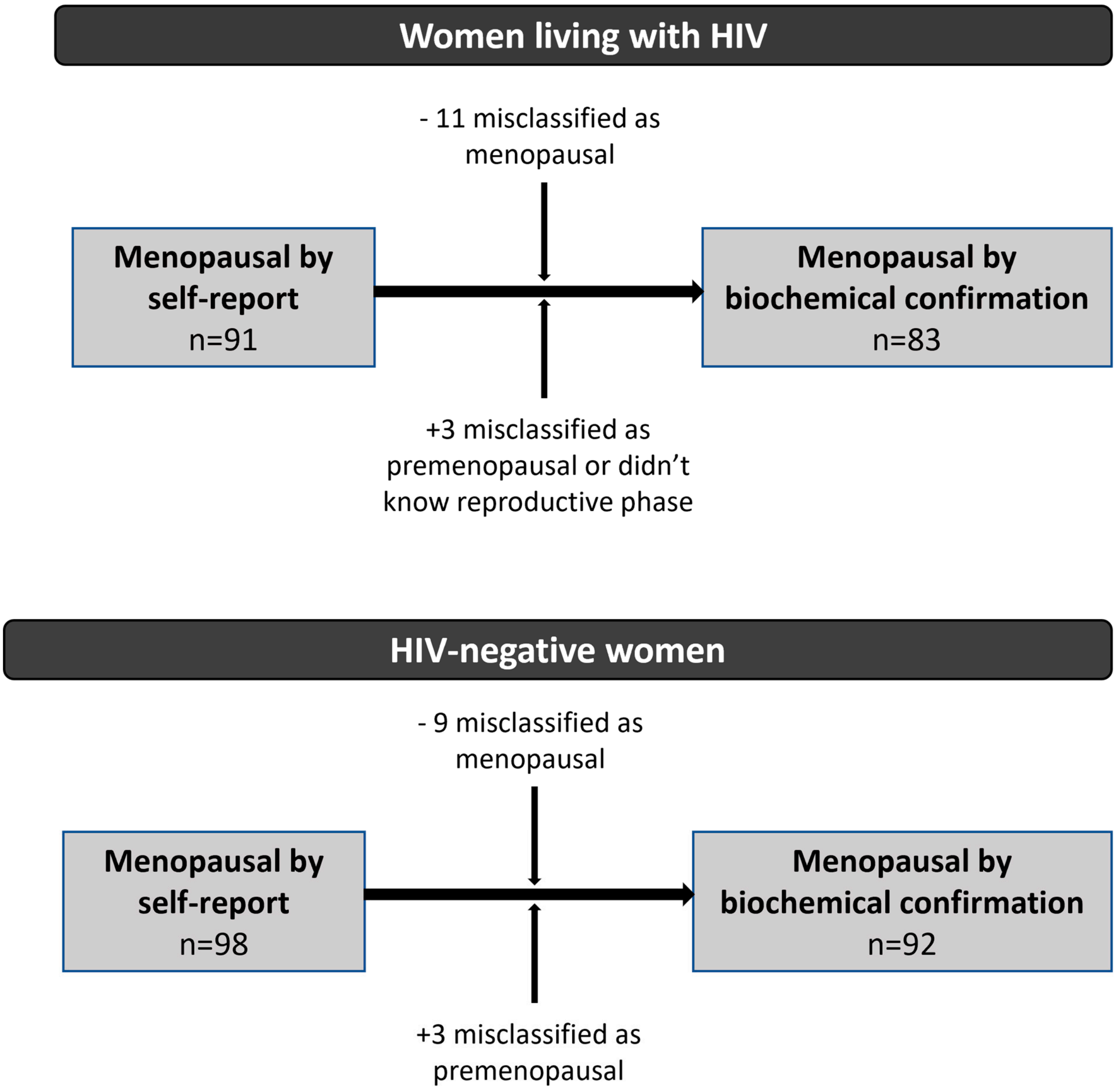

Figure S1. Flowchart of reproductive phase classification by self-report alone or self-report with biochemical confirmation based on follicle stimulating hormone levels
